# Bacterial Communities in Stream Biofilms in a Degrading Grassland Watershed on the Qinghai–Tibet Plateau

**DOI:** 10.3389/fmicb.2020.01021

**Published:** 2020-06-05

**Authors:** Ze Ren, Decao Niu, Panpan Ma, Ying Wang, Zhaomin Wang, Hua Fu, James J. Elser

**Affiliations:** ^1^State Key Laboratory of Grassland Agro-ecosystems, Key Laboratory of Grassland Livestock Industry Innovation, Ministry of Agriculture and Rural Affairs, Engineering Research Center of Grassland Industry, Ministry of Education, College of Pastoral Agriculture Science and Technology, Lanzhou University, Lanzhou, China; ^2^Flathead Lake Biological Station, University of Montana, Polson, MT, United States; ^3^Division of Biological Sciences, University of Montana, Missoula, MT, United States; ^4^Advanced Institute of Natural Sciences, Beijing Normal University, Zhuhai, China

**Keywords:** 16S rRNA, stream biofilm, normalized difference vegetation index, modularity, Qinghai–Tibet Plateau, grassland degradation

## Abstract

Grassland is among the largest terrestrial biomes and is experiencing serious degradation, especially on the Qinghai–Tibet Plateau (QTP). However, the influences of grassland degradation on microbial communities in stream biofilms are largely unknown. Using 16S rRNA gene sequencing, we investigated the bacterial communities in stream biofilms in sub-basins with different grassland status in the Qinghai Lake watershed. Grassland status in the sub-basins was quantified using the normalized difference vegetation index (NDVI). Proteobacteria, Bacteroidetes, Cyanobacteria, and Verrucomicrobia were the dominant bacterial phyla. OTUs, 7,050, were detected in total, within which 19 were abundant taxa, and 6,922 were rare taxa. Chao 1, the number of observed OTUs, and phylogenetic diversity had positive correlations with carbon (C), nitrogen (N), and/or phosphorus (P) in biofilms *per se*. The variation of bacterial communities in stream biofilms was closely associated with the rate of change in NDVI, pH, conductivity, as well as C, N, P, contents and C:N ratio of the biofilms. Abundant subcommunities were more influenced by environmental variables relative to the whole community and to rare subcommunities. These results suggest that the history of grassland degradation (indicated as the rate of change in NDVI) influences bacterial communities in stream biofilms. Moreover, the bacterial community network showed high modularity with five major modules (>50 nodes) that responded differently to environmental variables. According to the module structure, only one module connector and 12 module hubs were identified, suggesting high fragmentation of the network and considerable independence of the modules. Most of the keystone taxa were rare taxa, consistent with fragmentation of the network and with adverse consequences for bacterial community integrity and function in the biofilms. By documenting the properties of bacterial communities in stream biofilms in a degrading grassland watershed, our study adds to our knowledge of the potential influences of grassland degradation on aquatic ecosystems.

## Introduction

Landcover change, which has generally been considered a local environmental issue, is becoming a force of global importance with an outcome of degraded environmental conditions (Foley et al., 2005; Moges and Bhat, 2018). Owing to intimate connections between aquatic and terrestrial ecosystems (Williamson et al., 2008; Brett et al., 2017; Tanentzap et al., 2017), aquatic environments are strongly influenced by changes in surrounding landscapes (Vannote et al., 1980; Figueiredo et al., 2010; Dalu et al., 2018). As one of the largest terrestrial biomes in the world (Suttie et al., 2005), grasslands are facing widespread degradation (Gang et al., 2014), largely driven by anthropogenic activities (e.g., overgrazing and climate change) (Daily, 1995; Gang et al., 2014). Defined as a retrogressive ecosystem succession, grassland degradation has been demonstrated to disrupt aboveground vegetation (Deng et al., 2014; Wang et al., 2019) as well as soil physical, chemical, and biological properties (Viragh et al., 2011; Dlamini et al., 2014; Wang et al., 2020). These influences are likely transferred to adjacent aquatic ecosystems (Ren et al., 2019). Since grassland streams contribute approximately one-fifth of continental run-off on the Earth (Dodds, 1997), it is important to better understand the influences of grassland degradation on grassland streams.

As the primary receiver of nutrients and organic matter inputs from terrestrial ecosystems and the main channel linking upland watersheds with downstream aquatic ecosystems (Vannote et al., 1980; Cronan, 2012), streams play a critical role in biogeochemical processes of a watershed (Figueiredo et al., 2010; Du et al., 2020). Streams are strongly influenced by the landscapes through which they flow (Vannote et al., 1980; Ren et al., 2019) and, thus, are particularly sensitive to terrestrial disturbance (Holmes et al., 2012; Larson et al., 2019). The influences of changing landcover on adjacent aquatic ecosystems can usually be observed in the first place via responses of stream biofilms, which play an integral role in biogeochemical cycling in stream ecosystems through nutrient uptake, transfer of nutrients to higher trophic levels, and remineralization (Schiller et al., 2007; Battin et al., 2016; Stutter et al., 2020). Stream biofilms support complex microbial communities (Schiller et al., 2007; Battin et al., 2016), which are likely linked to watershed conditions because landcover controls the export of C, N, and P from terrestrial systems to streams (Erol and Randhir, 2013; Liu et al., 2019), potentially shifting *in situ* benthic biofilms.

Owing to the key roles of microbes in biogeochemical processes, assessment of microbial diversity is key for understanding the influences of external disturbances and environmental changes on aquatic ecosystems (Hosen et al., 2017). Previous studies have shown that microbial diversity in streams is affected by catchment landcover (Burgos-Caraballo et al., 2014). For example, by increasing stream water nutrients, urbanization has been shown to increase microbial diversity in stream biofilms (Burgos-Caraballo et al., 2014), although other studies have shown decreased microbial diversity because of increased nutrient loading and other anthropogenic disturbances (Kohler et al., 2011). Furthermore, Ren et al. (2019) has shown that grassland degradation differentially impacts inputs of N and P to streams, making it difficult to predict how microbial diversity would respond to grassland degradation.

Microbial processes in stream ecosystems are influenced by multiple environmental variables (Zeglin, 2015), such as nutrient availability (Levi et al., 2017; Qu et al., 2017), organic matter quantity and quality (Molinero and Burke, 2009; Mann et al., 2014), and hydrological factors (Valett et al., 1997; Ren et al., 2017a). In turn, these factors are determined by catchment landcover. Thus, it is reasonable to predict that microbial communities respond to landcover change due to the changes of these environmental variables. Previous studies have shown that microbial biomass and stoichiometry change significantly when landcover shifts from forest to agriculture or urban area (Teittinen et al., 2015; Qu et al., 2017). Our previous study has reported potential influence of grassland degradation on nutrient availabilities and limitation of stream biofilms (Ren et al., 2019). However, we still have very limited knowledge about the influences of grassland degradation on bacterial communities in these and similar streams that are affected by grassland deterioration.

Indeed, grassland degradation is a pervasive threat globally. Approximately 90% of the grassland in China has already been degraded due to a combination of anthropogenic and natural factors (Harris, 2010). On the Qinghai–Tibet Plateau (QTP), grassland is the dominant landcover and has undergone serious degradation in recent decades (Zhang et al., 2016; Ma et al., 2018). In degraded grassland systems, large losses of nutrients can occur due to erosion, loss of vegetation cover, and changes in soil texture (Dong et al., 2012; Su et al., 2015). Such impacts are likely associated with major changes in bacterial communities in adjacent streams. In this study, we investigated the bacterial communities in stream biofilms in the Qinghai Lake watershed on the northeast edge of the QTP within sub-catchments that are experiencing different degrees of degradation. Our aim was to document the structural properties of bacterial communities in stream biofilms and address the question: how do bacterial diversity and community respond to grassland degradation?

## Materials and Methods

### Sample Collection and Analyses

**FIGURE 1:**
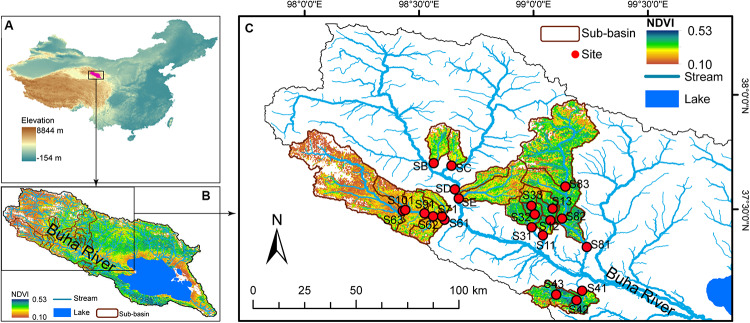
Study area and sample sites. **(A)** Qinghai Lake is the largest lake in China and is located on the northeast edge of the Qinghai–Tibet Plateau. **(B)** Buha river is the largest tributary of Qinghai Lake, which is an endorheic saline lake. **(C)** Water and benthic biofilm samples were collected at 22 sites. The map was cited from Ren (2019).

The study area was located on the northeast edge of the QTP (Figure 1), the Qinghai Lake Watershed (36°15′–38°20′ N and 97°50′–101°20′ E). Qinghai Lake Watershed is situated in a semiarid and cold climate zone with a high altitude from 3,194 to 5,174 m above sea level. The mean annual temperature is −0.7°C (Yi et al., 2010). The mean annual precipitation and evaporation are 363 mm and 1,300–2,000 mm, respectively, (Yi et al., 2010). As an endorheic saline lake, Qinghai Lake is the largest lake in China feeding by more than 40 tributaries, within which the Buha River is the largest (Figure 1). The watershed covers an area of 29,660 km^2^ of which 75% is covered by grassland. However, due to overgrazing, climate change, and other external disturbance, more than half of the grassland had been classified as degraded in 2010 (Luo et al., 2013).

Samples were collected from 22 stream sites in July 2017 (Figure 1). At each sample site, pH and conductivity (Cond) were measured *in situ* using a YSI handheld meter (model 80; YSI, Yellow Springs, OH, United States). Altitude was measured using a GPS unit (Triton 500; Magellan, Santa Clara, CA, United States). At each sampling point, six to nine submerged rocks were randomly chosen along the river cross section. The benthic biofilm was removed from the upper surface of each rock by rigorously brushing a 4.5-cm-diameter area with a sterilized nylon brush. The slurry was rinsed using sterile water. Approximately 10 ml of the mixed slurry was filtered onto a 0.2-μm polycarbonate membrane filter (Whatman, United Kingdom) that was immediately frozen in liquid nitrogen in the field. Twenty milliliters of mixed slurry was filtered onto pre-combusted glass fiber (GF/F) filters (six replicates) for later chemical analysis. Water samples, 500 ml, were also collected for chemical analyses.

Total nitrogen (TN) in the water samples was quantified by ion chromatography after persulfate oxidation (EPA 300.0). Total phosphorus (TP) was analyzed using the ascorbate acid colorimetric method after oxidation (EPA 365.3). DOC was analyzed on filtered (using pre-combusted GF/F filters) stream water using a Shimadzu TOC Analyzer (TOC-VCPH, Shimadzu Scientific Instruments, Columbia, MD, United States). Biofilm carbon (BFC) and nitrogen (BFN) were analyzed using an elemental analyzer (LECO 628). Biofilm phosphorus (BFP) was analyzed as soluble reactive phosphorus (SRP) after digestion with potassium persulfate (EPA 365.3).

### Normalized Difference Vegetation Index

Grassland status of the Qinghai Lake watershed was assessed using the normalized difference vegetation index (NDVI) based on Landsat images downloaded from USGS^1^ (Figure 1). As the most frequently used vegetation index, NDVI is commonly used to measure landcover status and monitor land degradation (Thiam, 2003; Yengoh et al., 2014). NDVI is calculated based on the absorption of red light and the reflection of infrared radiation by vegetation (Rouse et al., 1973). NDVI is calculated as (NIR-RED)/(NIR + RED), where NIR is near infrared reflectance and RED is visible red reflectance. Bare areas of soil, rock, and sand are represented by very low NDVI values (0∼0.1). Negative NDVI represents water bodies. NDVI has already been demonstrated to exhibit close relationships with above-ground vegetation biomass and coverage (Carlson and Ripley, 1997; Eastwood et al., 1997) and, thus, is commonly used to estimate regional vegetation coverage (Carlson and Ripley, 1997) and to indicate grassland degradation (Thiam, 2003; Li et al., 2013; Hilker et al., 2014). In this study, Landsat satellite images acquired in July 2017 and July 2009 were used to calculate NDVI in ArcGIS 14.0. The average NDVI of the sub-basin above the sample sites was calculated. Contemporary grassland status was represented by NDVI value in 2017 (shown as NDVI in the following analyses) and by the change rate of NDVI (calculated as the average annual change of NDVI from 2009 to 2017 and shown as NDVI.R in the following analyses). In Qinghai Lake watershed, NDVI had a large spatial variation and was closely associated with *in situ* measured vegetation status (e.g., aboveground biomass, grass species richness, and fractional coverage rate) but was not associated with altitude (Ren et al., 2019). Thus, using “space-for-time” substitution (Pickett, 1989; Blois et al., 2013), we could study the influences of grassland degradation on stream ecosystems according to spatial patterns of contemporary NDVI (Ren et al., 2019). The average annual change in NDVI from 2009 to 2017 was calculated as the rate of change in NDVI (indicated as NDVI.R in the following analyses).

### DNA Extraction, PCR, and Sequencing

DNA in stream biofilm samples was extracted using the TIANGEN-DP336 soil DNA Kit (TIANGEN-Biotech, Beijing, China) following the manufacturer’s recommendations. The forward primer 347F-CCTACGGRRBGCASCAGKVRVGAAT and reverse primer 802R-GGACTACNVGGGTWTCTAATCC (GENEWIZ, Inc., South Plainfield, NJ, United States) were used to amplify the V3 and V4 regions of the 16S rRNA genes. Samples were amplified using an initial denaturation of 3 min at 94°C, followed by 24 cycles of 30-s denaturation at 94°C, 90-s annealing at 57°C, 10-s extension at 72°C, and a final elongation for 10 min at 72°C. PCR products were verified on a 2% agarose gel and quantified using a Qubit 3.0 Fluorometer (Life Technologies, Germany). DNA libraries were sent to GENEWIZ, Inc., (Suzhou, China) for sequencing on an Illumina MiSeq platform (Illumina, San Diego, CA, United States).

### Sequence Analysis

The resulting 16S rRNA raw sequences (available at NCBI, PRJNA526110) were processed and analyzed with QIIME 1.9.1 (Caporaso et al., 2010). The *join_paired_ends.py* script was used to join forward and reverse reads. The paired-end sequences were assigned to samples based on barcode, trimmed by removing barcode and primer sequences, and quality filtered using *split_libraries.py* script. Low-quality sequences and the chimeric sequences were removed. Operational taxonomic units (OTUs) were clustered and assigned at 99% genetic similarity against the SILVA 132 database (Quast et al., 2013) using *pick_open_reference_otus.py* script. The *filter_otus_from_otu_table.py* script was used to remove the singletons, Archaea, chloroplast, and unknown OTUs (Kozich et al., 2013). To normalize surveying effort at the sample level, the *single_rarefaction.py* script was used with the depth of 25,884 sequence per sample (Supplementary Figure S1). The *alpha_diversity.py* script was used to calculate alpha diversity indices, including Chao 1, observed OTUs, Shannon, and PD whole tree.

### Statistical Analysis

Abundant and rare taxa were defined based on their relative abundances (Pedros-Alio, 2012; Logares et al., 2014). There are many publications that defined the rare and abundant taxa using different thresholds (Pedros-Alio, 2012; Logares et al., 2014; Liu et al., 2015; Chen et al., 2017; Xue et al., 2018; Zhang et al., 2018). In this study, OTUs were classified in different groups using the thresholds proposed by Liu et al. (2015), Chen et al. (2017), and Xue et al. (2018). OTUs with a relative abundance ≥1% in at least one sample and ≥0.01% in all samples were classified as abundant taxa (i.e., “AT” or “abundant subcommunity” hereafter). OTUs with a relative abundance <0.01% in at least one sample and <1% in all samples were classified as rare taxa (RT) and constituted the “rare subcommunity.” OTUs with a relative abundance ≥0.01% and <1% in all samples were classified as moderate taxa (MT). OTUs with a relative abundance <0.01% in at least one sample and ≥1% in at least one sample were classified as conditionally rare and abundant taxa (CRAT, as rare taxa in some samples but as abundant taxa in other samples).

The relationships between environmental variables and bacterial alpha diversity were assessed using Pearson correlation with stats v3.6.1 package (R Core Team, 2017). *P*-values of the mantel tests were adjusted using Benjamini and Hochberg (BH) methods (Benjamini and Hochberg, 1995). Bacterial community distributions along the NDVI gradient was assessed using non-metric multidimensional scaling (NMDS) with vegan 2.5-5 package (Oksanen et al., 2007). The relationships between environmental variables and bacterial community structure were assessed using distance-based redundancy analysis (dbRDA) with vegan 2.5-5 package (Oksanen et al., 2007). Furthermore, variation partitioning analyses (VPA) were performed to dissect the relative contributions of nutrients, NDVI, and other component factors to the variations of bacterial community structure.

The co-occurrence network of bacterial communities was established using R package igraph 1.2.4 (Csardi, 2013). Spearman correlations between all pairs of OTUs (OTUs presented in at least 11 samples and has an average relative abundance ≥0.01%) were calculated, and the *P*-values were adjusted using Benjamini and Hochberg (BH) methods (Benjamini and Hochberg, 1995). Only strong and significant correlations (*R* > 0.80 or *R* < -0.80, *P* < 0.05, post-BH correction) were considered and visualized. An Erdos–Renyi model was used to generate 99 random networks with the same size as the real networks. Topological parameters were calculated for both real and random networks. The *Z*-test was used to test the differences between the topological parameters of the real network and its random networks (Zhou et al., 2010; Kuang et al., 2017). Module structures and the connectivity [within-module connectivity (Zi) and among-module connectivity (Pi)] of each node (OTU) in the network were calculated. OTUs with Zi > 2.5 and Pi ≤ 0.62 were classified as “module hubs” (highly linked nodes within their own module). OTUs with Pi > 0.62 and Zi ≤ 2.5 were classified as “connectors” (nodes linking different modules). Mantel test and partial Mantel tests (controlling altitude) were conducted to assess the relationships between modules and environmental variables with vegan 2.5-5 package (Oksanen et al., 2007). As above, *P*-values were adjusted using Benjamini and Hochberg (BH) methods (Benjamini and Hochberg, 1995). All the analyses were conducted in R 3.4.1 (R Core Team, 2017).

## Results

### Alpha Diversity and Community Composition

**FIGURE 2:**
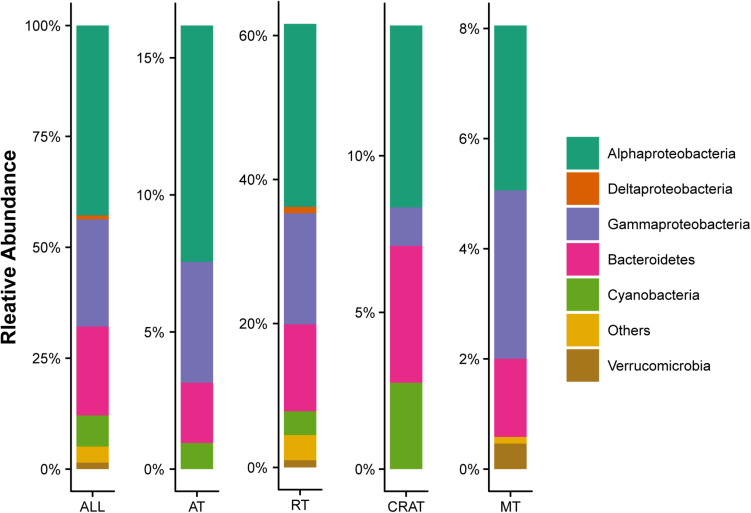
Composition structure of bacterial communities in stream biofilms. Only the dominant phyla with relative abundance >1% are shown. “All” represents the whole communities. “AT,” “RT,” “CRAT,” and “MT” represent subcommunities of abundant taxa, rare taxa, conditionally rare and abundant taxa, and moderate taxa, respectively.

In total, 7,050 OTUs were detected from 569,448 high-quality sequences at the 99% similarity level. Within the whole data set, 19 (0.3% of the total number) OTUs were classified as abundant (Supplementary Table S1), while 6,922 (98.2% of the total number) OTUs were rare, representing 16.2 and 61.6% of the total sequences, respectively, (Figure 2). Across all sample sites, the observed OTUs, Chao 1, Shannon diversity, and phylogenetic diversity were 3,299 ± 700, 4,400 ± 884, 9.41 ± 0.77, and 166 ± 27, respectively. Correlation analysis showed that the number of observed OTUs had significant positive relationships with BFC and BFP, Chao 1 had a significant positive relationship with BFP, and phylogenetic diversity had significant positive relationships with BFC, BFN, and BFP (Table 1). However, alpha diversity was not associated with NDVI, rate of change in NDVI, altitude, any of the tested physicochemical variables of stream water, nor the C:N:P ratios in stream water and biofilms.

**TABLE 1:** Correlation relationships between alpha diversity versus carbon, nitrogen, and phosphorus concentrations in stream biofilms.

	**Chao 1**		**Observed OTUs**		**Shannon**		**PD whole tree**	
	***R***	***p***	***r***	***p***	***R***	***p***	***R***	***p***
BFC	0.437	**0.042**	0.394	0.069	0.255	0.252	0.473	**0.026**
BFN	0.387	0.075	0.347	0.114	0.227	0.310	0.440	**0.040**
BFP	0.455	**0.033**	0.431	**0.045**	0.337	0.125	0.454	**0.034**

**P*-values are adjusted using Benjamini and Hochberg (BH) methods. Significant results (*P* < 0.05) are shown in bold.*

In bacterial communities in the stream biofilms, the dominant phyla (relative abundance >1%) were Proteobacteria (67.9%), followed by Bacteroidetes (20.1%) Cyanobacteria (7.0%), and Verrucomicrobia (1.5%) (Figure 2). Abundant taxa were affiliated to Alphaproteobacteria (8.6%, *n* = 11), Gammaproteobacteria (4.4%, *n* = 5), Bacteroidetes (2.2%, *n* = 2), and Cyanobacteria (1.0%, *n* = 1) (Figure 2 and Supplementary Table S1). The relative abundances of those bacterial phyla were not associated with environmental variables. As shown by the mean Bray–Curtis distance between all pairs of samples, abundant subcommunity had a significantly lower, while rare subcommunity had a significantly higher, mean Bray–Curtis distance value than the overall communities (Supplementary Figure S2), suggesting lower variabilities for abundant subcommunities but larger variabilities for rare subcommunities.

### Driving Factors of Spatial Patterns of Community Structure

**FIGURE 3:**
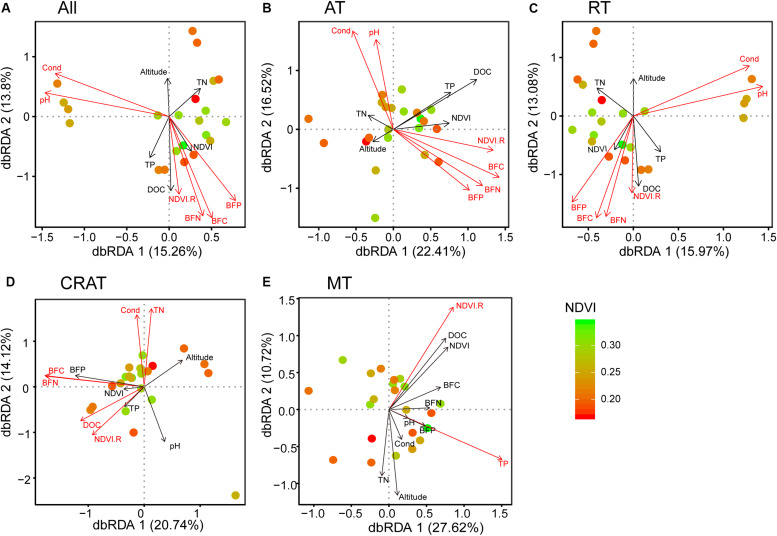
Distance-based redundancy analyses (dbRDA) reveal the relationships between bacterial communities (points) and environmental variables (arrows). Points are colored by normalized difference vegetation index (NDVI). Significant variables (*P* < 0.05 by envfit function) are shown in red. **(A)** “All,” **(B)** “AT,” **(C)** “RT,” **(D)** “CRAT,” and **(E)** “MT” represent the whole communities and the subcommunities of abundant taxa, rare taxa, conditionally rare and abundant taxa, and moderate taxa, respectively.

Distance-based redundancy analyses (dbRDA) showed that NDVI.R, pH, conductivity, as well as C, N, and P contents in stream biofilms had significant relationships (*P* < 0.05) with the distribution of the whole bacterial communities and the subcommunities of abundant taxa and rare taxa (Figure 3). NDVI.R, conductivity, DOC, TN, as well as C and N contents in stream biofilms had significant relationships (*P* < 0.05) with the distribution of the subcommunities of conditionally rare and abundant taxa (Figure 3D). NDVI.R and TP had significant relationships with the distribution of the subcommunities of moderate taxa (Figure 3E). The first two axes represented 29.1, 38.9, 29.1, 34.86, and 38.34% of the variances in terms of all, AT, RT, CRAT, and MT communities/subcommunities, respectively. Variation partitioning analyses (VPA) showed that the environmental variables explained 27.2, 37.8, 24.0, 35.3, and 26.9% of the compositional variances of these bacterial communities/subcommunities, respectively, (Supplementary Figure S3).

### Co-occurrence Network

**FIGURE 4:**
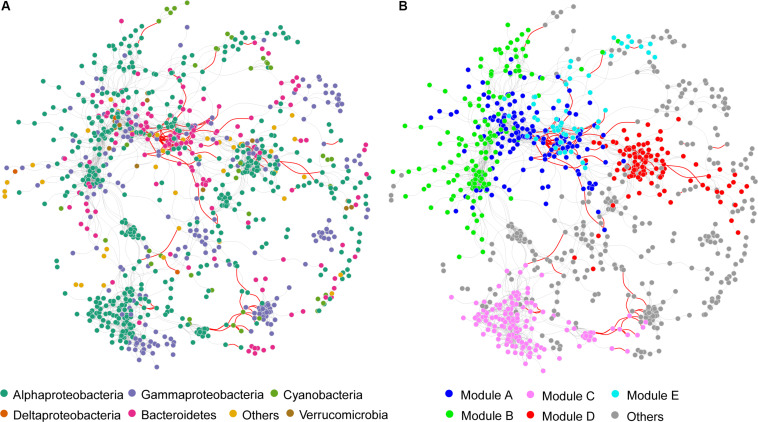
Co-occurrence networks of bacterial communities in stream biofilms. Nodes are colored by **(A)** taxonomic groups and **(B)** major modules (modules with more than 50 nodes). Edges represent Spearman’s correlation relationships, and only strong and significant relationships (Spearman *R* > 0.8, or *R* < -0.8, *P* < 0.05) are shown. *P*-values are adjusted using Benjamini and Hochberg (BH) methods. The gray lines indicate positive associations (co-occurrence interactions), and the red lines indicate negative associations (co-exclusion interactions). Circle nodes represent OTUs.

**FIGURE 5:**
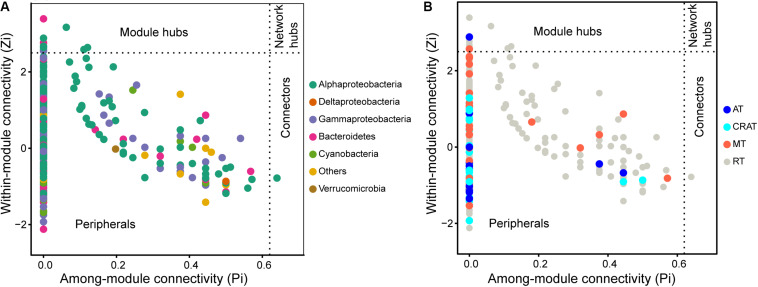
Topological roles of operational taxonomic units (OTUs) in the co-occurrence network according to within-module connectivity (Zi = 2.5) and among-module connectivity (Pi = 0.62). Each dot represents an OUT colored by **(A)** taxonomic groups and **(B)** categories defined by relative abundance. “All” represents the whole communities. “AT,” “CRAT,” “MT,” and “RT” represent subcommunities of abundant taxa, conditionally rare and abundant taxa, moderate taxa, and rare taxa, respectively. The detailed information of these connectors and module hubs are shown in Supplementary Table S2.

A co-occurrence network of bacterial communities in the stream biofilms was built based on pairwise correlation relationships between OTUs (Figure 4). The network was composed of 942 nodes connected by 3,206 edges (3.3% edges were negative correlations and 96.7% were positive). Topological parameters of the networks were calculated to describe the complex interrelationships between OTUs (Table 2). The distribution of node degree was well-fitted (*P* < 0.001) by the power law (Supplementary Figure S4), indicating that the network was scale-free and non-random. Moreover, the modularity, network centralization, clustering coefficient, and average path length of the real network were greater than those of its random network, suggesting that the bacterial network had “small world” property (any two nodes could interact with each other in the network through a few intermediaries) and significant modular structure (Figure 4 and Table 2). This network was clearly parsed into five major modules (modules with more than 50 nodes) (Figure 4 and Supplementary Figure S5). Mantel and partial Mantel tests showed close relationships between environmental variables and those major modules (Table 3). For example, modules A, B, and C were significantly associated with BFC, BFN, and BFP (Table 3). Modules B, C, and D were significantly associated with NDVI and NDVI.R (Table 3). According to the within-module connectivity (Zi) and between module connectivity (Pi), 12 module hubs and 1 module connector were identified (Figure 5 and Supplementary Table S2). The only module connector and 10 of the module hubs were rare taxa (Supplementary Table S2).

**TABLE 2:** Comparison of topological parameters between the network and its corresponding random network (*Z*-test).

**Topological Parameters**	**Description**	**Real**	**Random**
Number of nodes	Number of operational taxonomic units (OTUs) in the network	942	942
Number of edges	Strong and significant correlations	3206	3206
Negative edges	Negative correlations	105	105
Average degree	Average number of neighbors for all nodes	6.807	6.807
Average path length	Average of the shortest path lengths connecting each node to all other nodes	7.045	3.783 ± 0.006*
Clustering coefficient	Fraction of observed vs. possible clusters for each node	0.531	0.007 ± 0.001*
Centralization betweenness	The number of shortest paths between any two nodes in the graph passing through that node	0.09	0.012 ± 0.002*
Modularity	Tendency of a network to contain sub-clusters of nodes	0.827	0.355 ± 0.003*

*^∗^*P* < 0.05.*

**TABLE 3:** Correlation relationships between taxonomic dissimilarity of major modules and environmental variables.

**Environmental variables**	**Mantel *r***					**Partial Mental *r***				
	**Mod A**	**Mod B**	**Mod C**	**Mod D**	**Mod E**	**Mod A**	**Mod B**	**Mod C**	**Mod D**	**Mod E**
NDVI	−0.052	**−0.149**	**−0.116**	−0.109	−0.016	−0.085	**−0.213**	**−0.186**	**−0.159**	−0.101
NDVI.R	−0.031	**−0.132**	**−0.161**	**−0.159**	−0.096	−0.069	**−0.254**	**−0.249**	**−0.249**	**−0.236**
Altitude	−0.121	**−0.158**	−0.055	**−0.161**	**−0.211**	na	na	na	na	na
pH	0.078	0.085	**0.162**	**0.185**	0.055	0.043	0.125	**0.199**	**0.249**	0.046
Cond	0.089	**0.437**	**0.431**	0.185	**0.206**	0.060	**0.389**	**0.371**	0.101	0.125
DOC	0.024	−0.064	0.014	−0.122	−0.025	0.027	−0.108	−0.002	**−0.133**	−0.054
TN	−0.014	0.076	0.130	−0.024	−0.047	−0.010	0.117	0.117	−0.071	−0.104
TP	−0.067	−0.072	−0.091	−0.132	−0.035	−0.023	−0.116	−0.102	−0.093	0.004
BFC	**0.456**	**0.136**	**0.187**	0.147	0.091	**0.420**	**0.185**	**0.172**	0.119	0.060
BFN	**0.401**	0.077	**0.160**	0.112	−0.019	**0.388**	**0.143**	**0.158**	0.123	−0.023
BFP	**0.260**	0.058	0.109	0.079	−0.007	**0.286**	**0.137**	**0.136**	**0.134**	−0.009

*Spearman correlations (*r*) and associated *P*-values were calculated by Mantel test and partial Mantel test (controlling for altitude). *P*-values are adjusted using Benjamini and Hochberg (BH) methods. Significant correlations (*p* < 0.05) are shown in bold.*

## Discussion

In the biofilm bacterial communities of the QTP streams we sampled, the dominant phyla were Proteobacteria, Bacteroidetes, Cyanobacteria, and Verrucomicrobia, which are typical in freshwater ecosystems (Tamames et al., 2010). The relative abundances of these phyla were not associated with any of the tested environmental variables in our study. However, the variation of overall bacterial community composition in stream biofilms was significantly associated with the rate of change in NDVI, pH, conductivity, as well as C, N, P contents in the biofilm biomass. Many studies have shown that the properties of stream biofilms are influenced by activities in upstream catchments (Fanta et al., 2010; Qu et al., 2017). In grassland watersheds, large proportions of organic carbon and nutrient stocks are concentrated in the top soil layers (Gill et al., 1999; Du et al., 2019). External disturbances likely cause dramatic soil nutrient depletion and soil texture changes (Steffens et al., 2008; Dlamini et al., 2014; Wang et al., 2020). In degraded grassland ecosystems, increased soil erosion, loss of vegetation cover, and soil texture changes aggravate scour from surface flow, causing large losses of soil organic matter and nutrients to adjacent aquatic ecosystems (Dong et al., 2012; Su et al., 2015). This is of potential importance because terrestrial inputs are often the most important organic matter and nutrient sources, especially in small streams (Vannote et al., 1980; Meyer et al., 1981; Tank et al., 2010). These inputs can be accumulated and stored in benthic biofilms, influencing bacterial diversity, activities, and community compositions (Aubeneau et al., 2016; Shogren et al., 2020). Overall, our results are consistent with a previous study showing that diversity and composition of microbial communities in stream biofilms are more controlled by site-specific conditions than factors at the watershed scale (Burgos-Caraballo et al., 2014). Moreover, grassland degradation increases pH and influences free ion activities in soil (Viragh et al., 2011). These impacts could be transferred to adjacent stream ecosystems and affect stream water chemical conditions and, ultimately, their biological communities (Figueiredo et al., 2010; Zeglin, 2015). Thus, our results suggest that the history of grassland degradation (indicated as the rate of change in NDVI) might influence the bacterial communities in stream biofilms in this catchment, although the influence was not observed on relative abundances at the phylum level. These shifts in stream bacterial communities support a view of inland waters as sentinels and integrators of terrestrial processes due to their close connections via transport and storage of water, nutrients, and energy.

In our studied bacterial communities, more than 98% OTUs were rare taxa that accounted for 61.6% of the total sequences, while only 0.3% OTUs were abundant but accounting for 16.2% of the total sequences. Biological communities normally contain a small number of abundant taxa and many rare species (Magurran and Henderson, 2003). This is also true of microbial communities, for which most taxa are extremely rare (Logares et al., 2014). Abundant and rare microbial subcommunities may have intrinsic characteristics that result in different contributions to community dynamics and ecosystem functions (Logares et al., 2014; Xue et al., 2018). Abundant taxa are the core taxa that contribute the most ecosystem functions, such as nutrient cycling, biomass production, and energy flow, and are maintained because of their high growth rate and close adaptation to particular habitats (Pedros-Alio, 2006; Jiao et al., 2019). Drastic variations in abundant taxa could cause significant effects on ecosystem processes (Logares et al., 2014; Rivett and Bell, 2018). In contrast, rare taxa may have low growth rates but encompass a broad diversity, explaining large amounts of microbial community dynamics and dissimilarities (Pedros-Alio, 2012; Ruiz-Gonzalez et al., 2019). It has been proposed that rare taxa include ecologically redundant taxa that could maintain ecosystem functioning and contribute to community stability during environmental perturbations that impact dominant taxa (Pedros-Alio, 2012). In our study, the rare subcommunities showed similar associations with environmental variables as the whole communities, while the abundant subcommunities were more affected by those environmental variables (Figure 3). Thus, we infer that grassland degradation and associated environmental changes influence stream ecosystem functions via more intense effects on abundant subcommunities than that on rare subcommunities.

Microbial communities are typically complex and composed of taxa with potential for strong interactions. In these highly complex systems, co-occurrence patterns are important in understanding microbial community structure by offering new insights into potential biotic interactions and community assembly mechanisms (Fuhrman, 2009; Faust and Raes, 2012; Ren and Gao, 2019). Modularity is a characteristic of most large complex systems, representing the tendency to contain sub-clusters of members in a network (Newman, 2006) and is usually related to habitat heterogeneity and niche diversity across broad scales (Barberan et al., 2012; Ren et al., 2017b). The sub-cluster or module is a group of highly interconnected species with fewer connections with other groups (Newman, 2006). Module structures can reveal more ecological properties of complex communities than studying the communities in taxonomic groups or as a whole (Thompson, 2005). Thus, the relationships between microbial community modules and environmental variables can improve our understanding of the influences of environmental variation on microbial community assembly (Menezes et al., 2015; Toju et al., 2016). Our data showed that the bacterial community network in the grassland watershed of Qinghai Lake had a high modularity with five major modules (module with more than 50 nodes), which had different taxonomic composition (Supplementary Figure S5). Module-A was dominated by Bacteroidetes in abundance. Module-B, C, and D were dominated by Alphaproteobacteria in abundance. Module-E was dominated by Gammaproteobacteria in abundance. The modules also responded differently to environmental variables (Table 3). For example, module-A, B, and C were closely associated with C, N, and P in stream biofilms. Module-B, C, and D were closely associated with NDVI and the rate of change in NDVI, as well as stream water pH and conductivity. Module-E only associated with the rate of change in NDVI. According to module structure, some nodes are identified as keystone taxa indicating their important roles in structuring network and maintaining ecosystem stability (Banerjee et al., 2018). “Connectors” are the nodes linking different modules. “Module hubs” are the nodes highly linked with others within a module. The loss of those keystone taxa will increase network fragmentation (Widder et al., 2014; Banerjee et al., 2018). In our bacterial network, there was only one “connector” identified (*Pseudorhodobacter*, classified as a rare taxon), suggesting high fragmentation of the network and strong independence of the modules. Moreover, there were 12 “module hubs,” 10 of which were rare taxa (Supplementary Table S2). These rare taxa are easily affected by various disturbances due to their weaker competitive capabilities and low growth rates (Logares et al., 2015). The loss of these keystone but rare taxa may result in a more fragmented network and cause adverse consequences for bacterial community integrity and function in stream biofilms.

Stream biofilms are very complex systems composed of various organisms, including bacteria, algae, fungi, protozoa, and others (Battin et al., 2016). These organisms can have strong interactions involving both cooperation and competition (Fitter and Hillebrand, 2009; Rendueles and Ghigo, 2015) that are driven by a large number of environmental factors, including, but not limited to, nutrient availability (Tank and Dodds, 2003; Myrstener et al., 2018), temperature (Zhao et al., 2018), light (Fanta et al., 2010; Zhao et al., 2018), hydrological factors (Valett et al., 1997; Ren et al., 2017a), and land use (Burgos-Caraballo et al., 2014; Qu et al., 2017). Even terrestrial bacteria can contribute to the complexity of bacterial communities in stream biofilms (Ruiz-Gonzalez et al., 2015; Caillon and Schelker, 2020). Our data indicate that these interactions can be strongly affected by grassland degradation in the QTP. We acknowledge that there are limitations in our interpretation, which are based on limited spatiotemporal scales, a subset of potential environmental variables, and a subset of organisms without considering eukaryotic algae, fungi, protozoa, etc. More studies, including experimental manipulations and other analyses technologies, are needed. For example, more streams and longer-term investigations will be helpful in alleviating stochastic influences. Inclusion of more environmental variables, such as hydrological, geological, and climatic factors, may also help explain community variations. Controlled experiments will also help reveal the underlying mechanisms of community response to grassland degradation. However, we believe our study has added useful knowledge about how bacterial communities in stream biofilms respond in a watershed undergoing grassland degradation.

## Data Availability Statement

The datasets generated for this study can be found in the NCBI, PRJNA526110.

## Author Contributions

ZR, DN, HF, and JE designed the study. ZR, YW, PM, and ZW performed the field work and the laboratory analysis. ZR analyzed the data and prepared the manuscript. All authors reviewed the manuscript.

## Conflict of Interest

The authors declare that the research was conducted in the absence of any commercial or financial relationships that could be construed as a potential conflict of interest.

## Footnotes

1 https://earthexplorer.usgs.gov/
