# Supplementary material for: Bacterial Communities in Stream Biofilms in a Degrading Grassland Watershed on the Qinghai–Tibet Plateau
Source: Front Microbiol. 2020 Jun 5;11:1021. doi: 10.3389/fmicb.2020.01021 (PMC7290132; doi:10.3389/fmicb.2020.01021)
Supplement: Supplementary file 1 [file Data_Sheet_1.docx]

# Bacterial communities in stream biofilms in a degrading grassland watershed on the Qinghai-Tibet Plateau

Ze Ren^a,b,c,d*^, Decao Niu^a^, Panpan Ma^a^, Ying Wang^a^, Zhaomin Wang^a^, Hua Fu^a^, James J. Elser^b,c*^

^a^ State Key Laboratory of Grassland Agro-ecosystems; Key Laboratory of Grassland Livestock Industry Innovation, Ministry of Agriculture and Rural Affairs; Engineering Research Center of Grassland Industry, Ministry of Education; College of Pastoral Agriculture Science and Technology, Lanzhou University, Lanzhou, 730020, China

^b^ Flathead Lake Biological Station, University of Montana, Polson, MT 59860, USA

^c^ Division of Biological Sciences, University of Montana, Missoula, MT 59812, USA

^d^ Advanced Institute of Natural Sciences, Beijing Normal University at Zhuhai, Zhuhai, 519087, China

***Corresponding Authors**:

**Ze Ren**: [renzedyk@gmail.com](mailto:renzedyk@gmail.com), Division of Biological Sciences, University of Montana, Missoula, MT 59812, USA

**James J. Elser**: [jim.elser@flbs.umt.edu](mailto:jim.elser@flbs.umt.edu), Flathead Lake Biological Station, University of Montana, Polson, MT 59860, USA

## Supplementary Information

Figure S1 Rarefaction curves of bacterial communities in stream biofilms. Curves are colored by sample sites.


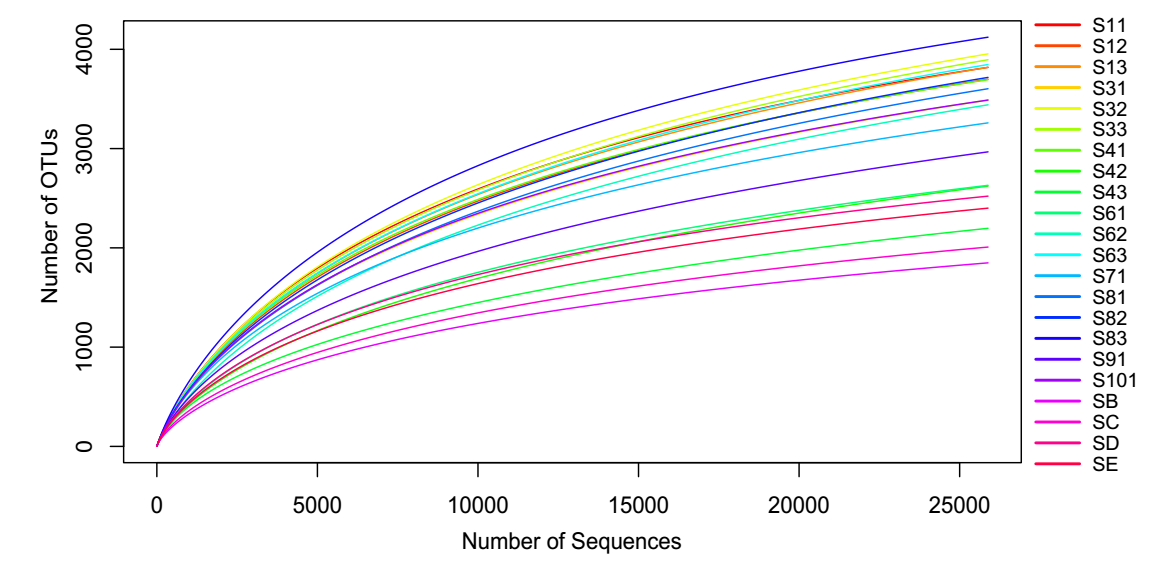


Figure S2 Box plot of pairwise Bray-Curtis distances of bacterial communities in stream biofilms. OTUs categories are defined by relative abundance. The top, middle, and bottom lines of the boxes represent the 25^th^, 50^th^, and 75^th^ percentiles of the value. Different letters above each box indicate significant differences (ANOVA, P<0.05). “All” represents the whole communities. “AT”, “CRAT”, “MT”, and “RT” represent subcommunities of abundant taxa, conditionally rare and abundant taxa, moderate taxa, and rare taxa, respectively.


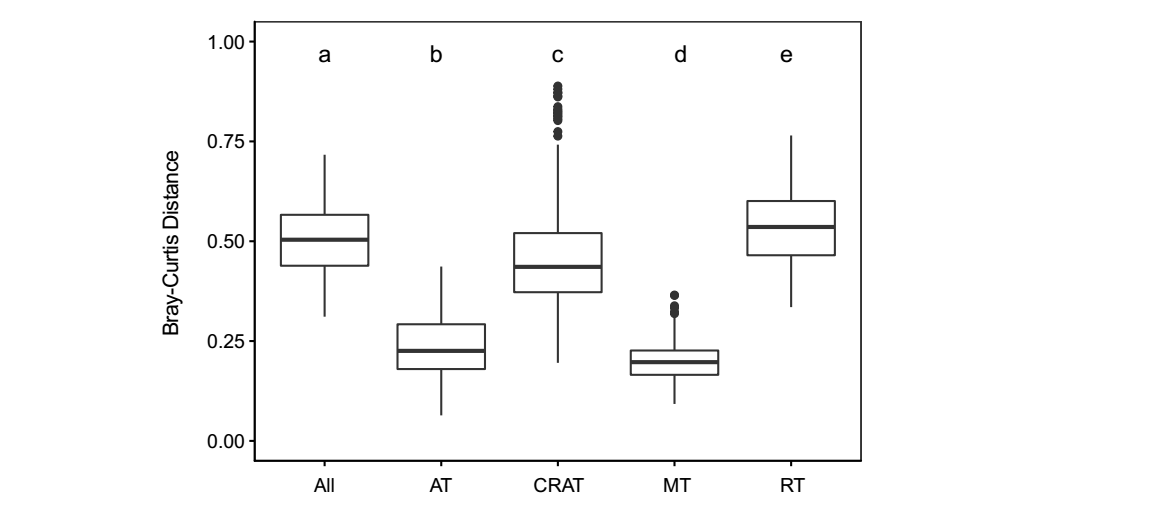


Figure S3 Variance partition analysis (VPA) determined the relative contributions of three group factors and the interactions between two or three of the factors. These three group factors are vegetation (NDVI and NDVI.R), nutrients (DOC, TN, TP, BFC, BFN, and BFP), and others (conductivity, altitude, and pH). The relative variance proportions that the corresponding components could explain are shown in percentages. “All” represents the whole communities. “AT”, “RT”, “CRAT”, and “MT” represent subcommunities of abundant taxa, rare taxa, conditionally rare and abundant taxa, and moderate taxa, respectively.


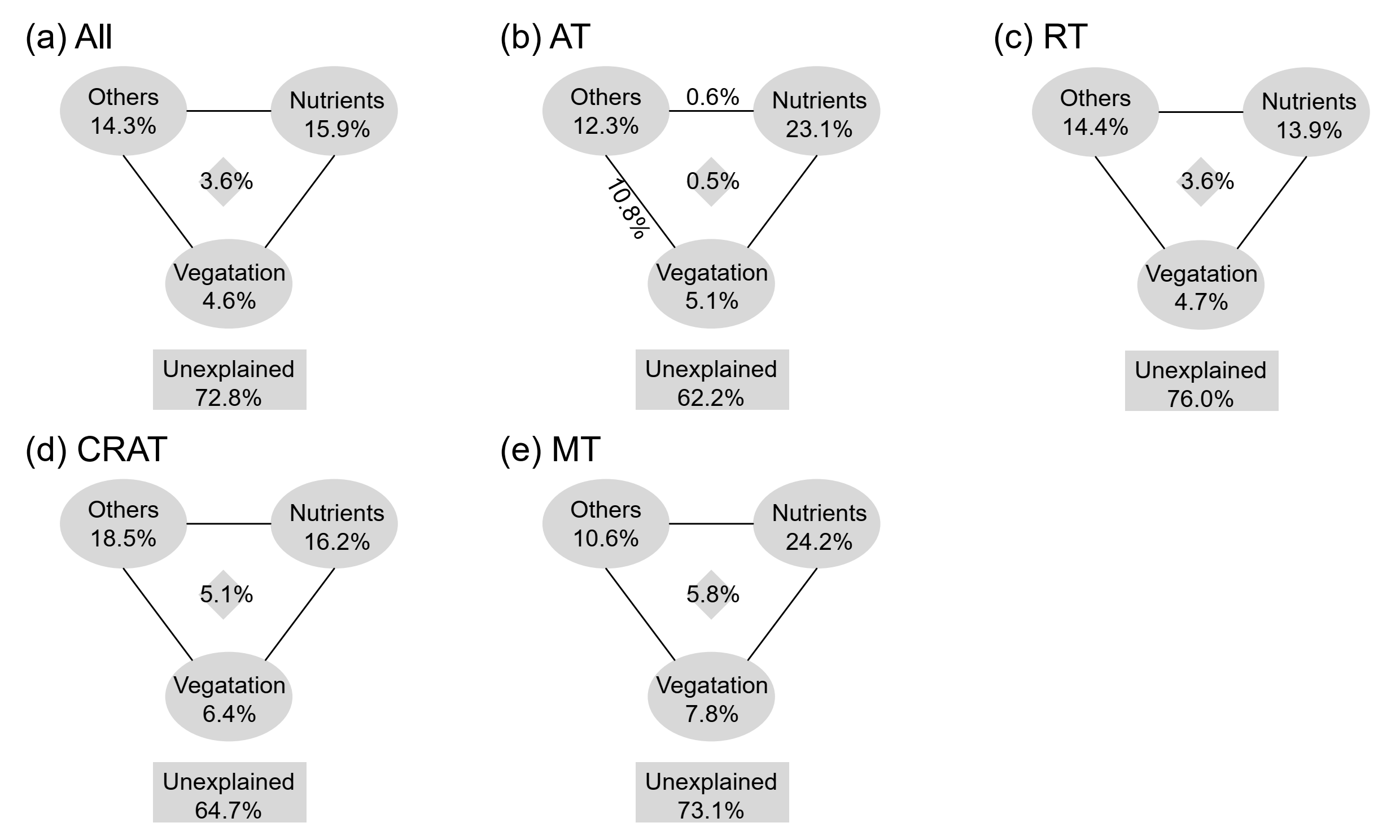


Figure S4 The node degree distributions of real network (blue) and random networks (grey) of bacterial communities in stream biofilms.


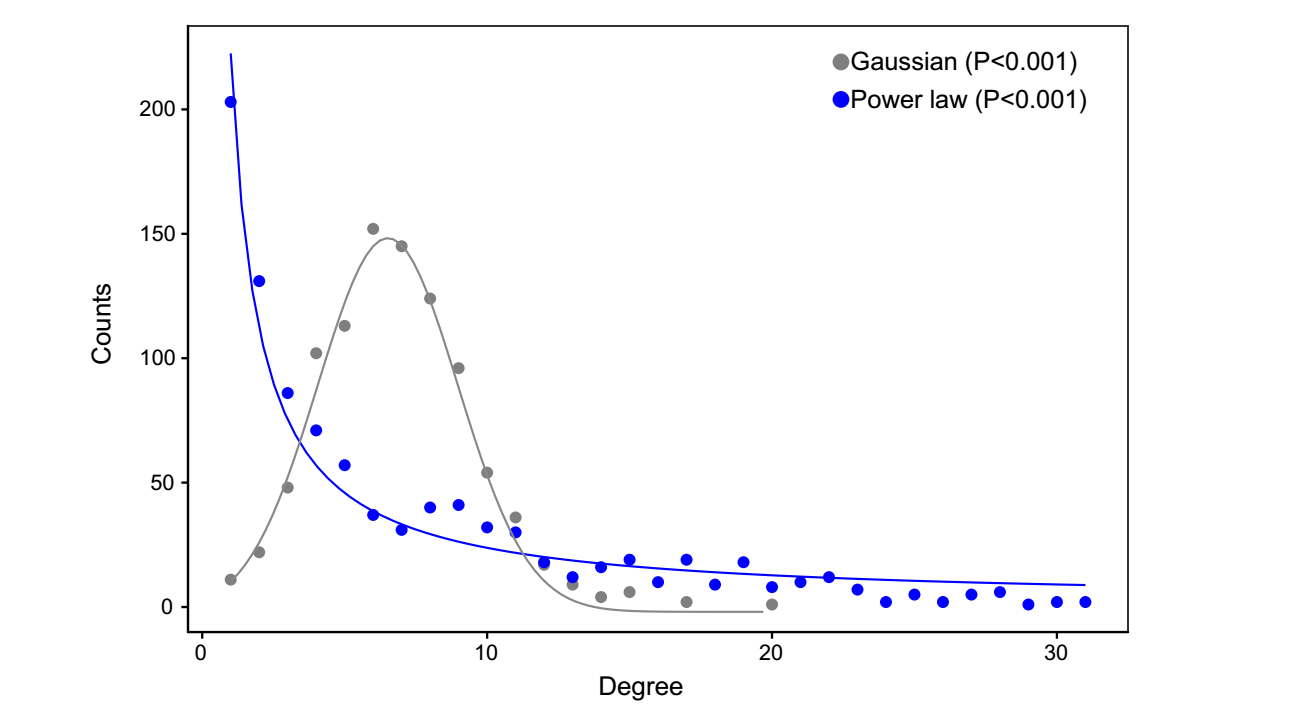


Figure S5 Taxonomic composition of the network modules shown in (a) relative abundance (%) Rand (b) number of OTUs.


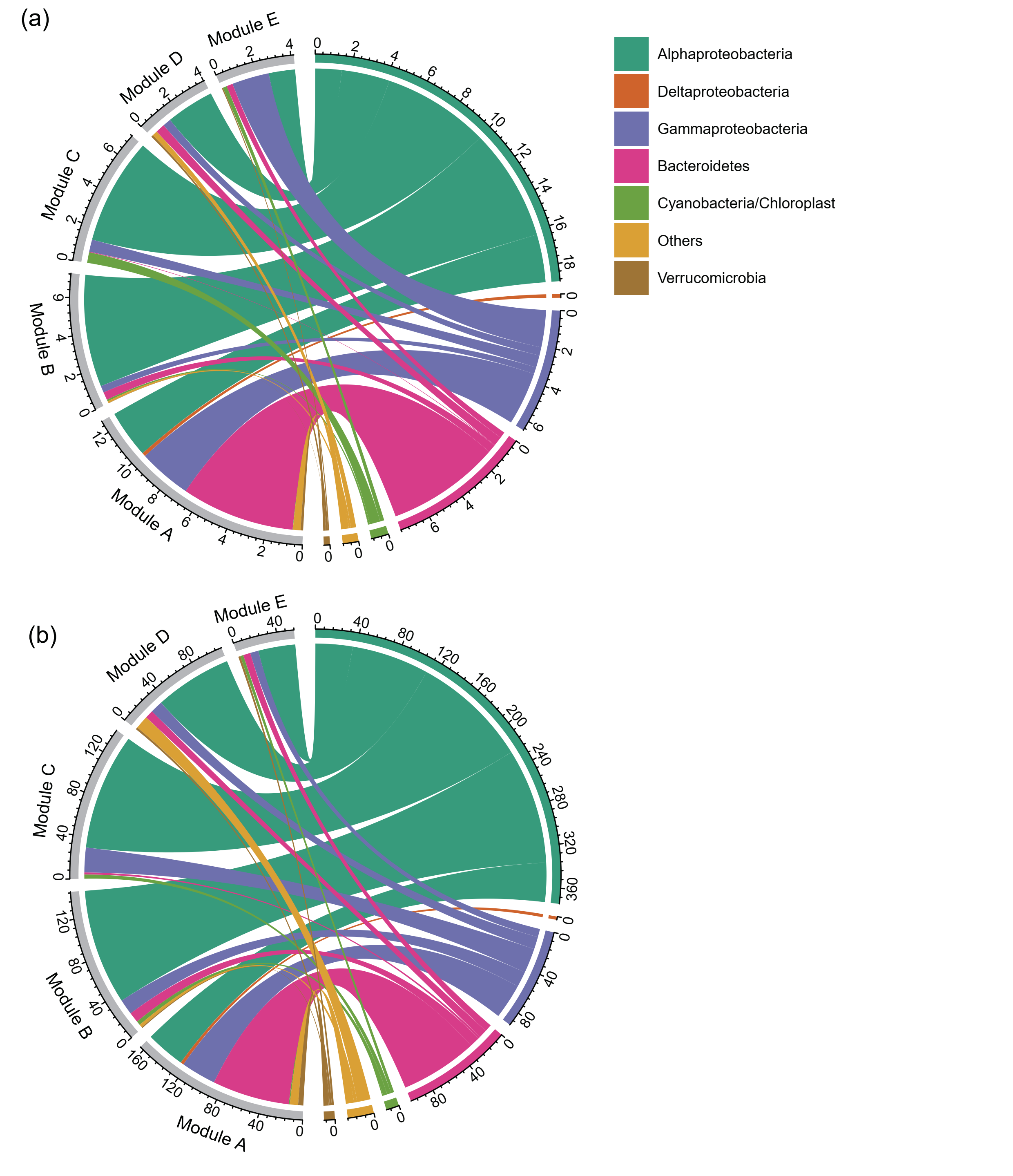


Table S1 Taxonomic information of the abundant taxa

| OTU ID | Relative  Abundance | Taxonomy |
| --- | --- | --- |
| otu357022 | 0.36% | D0_Bacteria; D1_Bacteroidetes; D2_Bacteroidia; D3_Chitinophagales; D4_Chitinophagaceae; D5_Ferruginibacter; D6_uncultured bacterium |
| otu192389 | 1.83% | D0_Bacteria; D1_Bacteroidetes; D2_Bacteroidia; D3_Cytophagales; D4_Spirosomaceae; D5_Arcicella; D6_uncultured bacterium |
| otu8348 | 0.95% | D0_Bacteria; D1_Cyanobacteria; D2_Oxyphotobacteria; D3_Nostocales; D4_Phormidiaceae; D5_Tychonema CCAP 1459-11B |
| otu91364 | 0.63% | D0_Bacteria; D1_Proteobacteria; D2_Alphaproteobacteria; D3_Caulobacterales; D4_Caulobacteraceae; D5_Brevundimonas |
| otu235616 | 0.29% | D0_Bacteria; D1_Proteobacteria; D2_Alphaproteobacteria; D3_Rhodobacterales; D4_Rhodobacteraceae; D5_Pseudorhodobacter |
| otu72322 | 1.22% | D0_Bacteria; D1_Proteobacteria; D2_Alphaproteobacteria; D3_Rhodobacterales; D4_Rhodobacteraceae; D5_Pseudorhodobacter; D6_uncultured bacterium |
| otu54665 | 0.97% | D0_Bacteria; D1_Proteobacteria; D2_Alphaproteobacteria; D3_Rhodobacterales; D4_Rhodobacteraceae; D5_Pseudorhodobacter; D6_uncultured bacterium |
| otu76083 | 0.85% | D0_Bacteria; D1_Proteobacteria; D2_Alphaproteobacteria; D3_Sphingomonadales; D4_Sphingomonadaceae; D5_Polymorphobacter |
| otu85345 | 0.57% | D0_Bacteria; D1_Proteobacteria; D2_Alphaproteobacteria; D3_Sphingomonadales; D4_Sphingomonadaceae; D5_Sphingomonas |
| otu270397 | 0.35% | D0_Bacteria; D1_Proteobacteria; D2_Alphaproteobacteria; D3_Sphingomonadales; D4_Sphingomonadaceae; D5_Sphingomonas; D6_uncultured bacterium |
| otu354354 | 0.62% | D0_Bacteria; D1_Proteobacteria; D2_Alphaproteobacteria; D3_Sphingomonadales; D4_Sphingomonadaceae; D5_Sphingorhabdus |
| otu288327 | 1.82% | D0_Bacteria; D1_Proteobacteria; D2_Alphaproteobacteria; D3_Sphingomonadales; D4_Sphingomonadaceae; D5_Sphingorhabdus; D6_uncultured bacterium |
| otu93766 | 0.76% | D0_Bacteria; D1_Proteobacteria; D2_Alphaproteobacteria; D3_Sphingomonadales; D4_Sphingomonadaceae; D5_Sphingorhabdus; D6_uncultured bacterium |
| otu349146 | 0.55% | D0_Bacteria; D1_Proteobacteria; D2_Alphaproteobacteria; D3_Sphingomonadales; D4_Sphingomonadaceae; D5_uncultured; D6_uncultured bacterium |
| otu103015 | 1.01% | D0_Bacteria; D1_Proteobacteria; D2_Gammaproteobacteria; D3_Betaproteobacteriales; D4_Burkholderiaceae; D5_Hydrogenophaga |
| otu351024 | 0.71% | D0_Bacteria; D1_Proteobacteria; D2_Gammaproteobacteria; D3_Betaproteobacteriales; D4_Burkholderiaceae; D5_Hydrogenophaga |
| otu222488 | 0.59% | D0_Bacteria; D1_Proteobacteria; D2_Gammaproteobacteria; D3_Betaproteobacteriales; D4_Burkholderiaceae; D5_Polaromonas |
| otu136842 | 1.80% | D0_Bacteria; D1_Proteobacteria; D2_Gammaproteobacteria; D3_Betaproteobacteriales; D4_Burkholderiaceae; D5_Polaromonas; D6_uncultured bacterium |
| otu115425 | 0.30% | D0_Bacteria; D1_Proteobacteria; D2_Gammaproteobacteria; D3_Betaproteobacteriales; D4_Burkholderiaceae; D5_Rhodoferax |

Table S2 List of module hubs and connectors in co-occurrence networks according to the connectivity of each node. In category II, “CRAT”, “MT”, and “RT” represent subcommunities of conditionally rare and abundant taxa, moderate taxa, and rare taxa, respectively.

| OTU ID | Pi | Zi | Module | Category I | Category II | Taxonomy |
| --- | --- | --- | --- | --- | --- | --- |
| otu368268 | 0.000 | 3.391 | Module A | Module Hubs | RT | D0_Bacteria; D1_Bacteroidetes; D2_Bacteroidia; D3_Cytophagales; D4_Spirosomaceae; D5_Arcicella; D6_uncultured bacterium |
| otu335993 | 0.062 | 3.169 | Module D | Module Hubs | RT | D0_Bacteria; D1_Proteobacteria; D2_Alphaproteobacteria; D3_Sphingomonadales; D4_Sphingomonadaceae; D5_Sphingorhabdus; D6_uncultured bacterium |
| otu270397 | 0.000 | 2.885 | Module D | Module Hubs | RT | D0_Bacteria; D1_Proteobacteria; D2_Alphaproteobacteria; D3_Sphingomonadales; D4_Sphingomonadaceae; D5_Sphingomonas; D6_uncultured bacterium |
| otu121247 | 0.000 | 2.766 | Module B | Module Hubs | RT | D0_Bacteria; D1_Proteobacteria; D2_Alphaproteobacteria; D3_Sphingomonadales; D4_Sphingomonadaceae; D5_Rhizorhapis; D6_uncultured bacterium |
| otu249366 | 0.000 | 2.760 | Module A | Module Hubs | RT | D0_Bacteria; D1_Bacteroidetes; D2_Bacteroidia; D3_Cytophagales; D4_Spirosomaceae; D5_Arcicella; D6_uncultured bacterium |
| otu256858 | 0.000 | 2.760 | Module A | Module Hubs | MT | D0_Bacteria; D1_Bacteroidetes; D2_Bacteroidia; D3_Cytophagales; D4_Spirosomaceae; D5_Arcicella; D6_uncultured bacterium |
| otu4166 | 0.000 | 2.674 | Module C | Module Hubs | RT | D0_Bacteria; D1_Proteobacteria; D2_Alphaproteobacteria; D3_Sphingomonadales; D4_Sphingomonadaceae; D5_Porphyrobacter; D6_uncultured bacterium |
| otu119154 | 0.121 | 2.639 | Module B | Module Hubs | RT | D0_Bacteria; D1_Proteobacteria; D2_Alphaproteobacteria; D3_Sphingomonadales; D4_Sphingomonadaceae; D5_Rhizorhapis; D6_uncultured bacterium |
| otu414134 | 0.000 | 2.639 | Module B | Module Hubs | RT | D0_Bacteria; D1_Proteobacteria; D2_Alphaproteobacteria; D3_Sphingomonadales; D4_Sphingomonadaceae; D5_Rhizorhapis; D6_uncultured bacterium |
| otu175808 | 0.111 | 2.588 | Module E | Module Hubs | RT | D0_Bacteria; D1_Proteobacteria; D2_Alphaproteobacteria; D3_Caulobacterales; D4_Caulobacteraceae; D5_Brevundimonas |
| otu170678 | 0.000 | 2.570 | Others | Module Hubs | RT | D0_Bacteria; D1_Proteobacteria; D2_Alphaproteobacteria; D3_Sphingomonadales; D4_Sphingomonadaceae; D5_Polymorphobacter |
| otu256408 | 0.000 | 2.550 | Module A | Module Hubs | CRAT | D0_Bacteria; D1_Proteobacteria; D2_Gammaproteobacteria; D3_Betaproteobacteriales; D4_Burkholderiaceae; D5_Leptothrix |
| otu77500 | 0.640 | -0.781 | Module B | Connectors | RT | D0_Bacteria; D1_Proteobacteria; D2_Alphaproteobacteria; D3_Rhodobacterales; D4_Rhodobacteraceae; D5_Pseudorhodobacter |
